# Achieving Equity in Child and Adolescent Mental Health by Addressing Racism Through Prevention Science

**DOI:** 10.1007/s42844-023-00104-1

**Published:** 2023-08-22

**Authors:** Nada M. Goodrum, Daniel K. Cooper, Sarah Edmunds, Guillermo M. Wippold, Jessica Bradshaw, Julie K. Nguyen, Norweeta Milburn, Funlola Are

**Affiliations:** 1 Department of Psychology, University of South Carolina, 1512 Pendleton Street, Barnwell College, Suite #220, Columbia, SC 29208, USA; 2 Department of Psychiatry and Biobehavioral Sciences, University of California, Los Angeles, CA, USA; 3 Louis A. Faillace, M.D., Department of Psychiatry and Behavioral Sciences, University of Texas Health Science Center, Houston, TX, USA

**Keywords:** Equity, Racism, Child mental health, Prevention science, Prevention

## Abstract

Prevention science is a multidisciplinary field dedicated to promoting public health and reducing early risk factors that lead to negative health outcomes. It has been used to successfully improve child and family mental health and well-being, including for families affected by adversity. Despite advances in prevention efforts, major public health inequities remain for Black, Indigenous, and other People of Color (BIPOC) children and families, in part because of equity-implicit “one-size-fits-all” approaches that do not directly address racism which in part underlies the very health concerns these efforts aim to prevent. Structural racism not only introduces additional risk for negative health outcomes for BIPOC families but also it reduces access to prevention-focused programs and policies, thus perpetuating inequities across generations. Adopting an equity-explicit, antiracist lens that attends to the effects of structural racism can strengthen the impact of prevention efforts by more effectively improving child and family health, reducing access barriers, and effecting multigenerational change for BIPOC families experiencing various levels of adversity. Evidence-informed recommendations for applying antiracist prevention science include the following: explicitly incorporating an understanding of structural racism within prevention science methods and theory (e.g., risk and resilience frameworks); establishing and fostering truly equitable community partnerships; diversifying the field through mentorship of BIPOC scholars and clinicians focused on child and family well-being; assembling diverse transdisciplinary research teams to address child health inequities in a family-centered manner; attending to intersectionality; and using implementation science to promote access and sustainability for all families.

Despite half a century of academic research documenting the detrimental health effects of racism (Williams et al., 2019), it was only recently—in 2021—that the Centers for Disease Control and Prevention (CDC) formally recognized structural racism as a public health threat in the USA (Centers for Disease Control and Prevention, 2021). *Structural racism* refers to a system of policies, institutions, laws, and cultural norms that reinforce racial hierarchies based on a history of White supremacy (Trent et al., 2019; Williams et al., 2019), and encompasses multiple levels and systems (interpersonal, institutional, cultural, etc.). Consistent with an intersectionality framework, structural racism works in tandem with other interlocking forms of oppression. Although it is well known that structural racism and its effects have created and maintained significant inequities in children’s mental health, most prevention-focused programs to support children’s mental health adopt a colorblind, “one-size-fits-all” approach that does not directly address racism. The field of prevention science is ideally suited to promote health equity and improve the lives of children and families of color, yet this potential is not being fully realized due to the preponderance of equity-implicit approaches (i.e., approaches that do not directly address racism to help ensure that equity can be achieved). We argue that achieving racial equity in child and adolescent mental health requires equity-explicit approaches that directly consider and address structural racism and its effects.

## Prevention Science and Persisting Mental Health Disparities

Prevention science is a multidisciplinary field that aims to promote public health by reducing early risk factors and leveraging protective factors that are related to positive health outcomes (Catalano et al., 2012; Fishbein, 2021). Prevention science has been used to study and disseminate interventions that promote positive child and family health outcomes. For example, family-based preventive interventions have been successful in reducing children’s internalizing problems, externalizing problems, substance use, and other risky behaviors as well as promoting positive outcomes including children’s self-regulation, academic functioning, and physical health (Sandler et al., 2015). Programming with a family-centered approach (i.e., that empowers families to make decisions about the supports they receive) has long been shown to enhance child outcomes (Dunst, 2002). Family-based programs can also prevent or mitigate the occurrence and impact of traumatic stress, including racism-related traumatic stress, in children (Goodrum & Prinz, 2022; Metzger et al., 2021). Beyond effects on child mental health and family functioning, a few family-based prevention programs have even demonstrated effects on families’ economic situations (Patterson et al., 2010), including generational effects where children receiving a prevention program had higher earnings 20 years later as adults (Gertler et al., 2013).

Despite these successes in prevention efforts, behavioral health problems remain prevalent for BIPOC youth and racial inequities persist. For example, suicide rates recently declined among white youth but increased among Black and Asian American/Pacific Islander youth (Ramchand et al., 2021). Meanwhile, as a result of discriminatory policies, institutions, and laws, racial disparities in accessing mental health services worsened between 2010 and 2017 (Rodgers et al., 2022). White youths access mental health services at nearly double the rate of Black and Hispanic/Latinx youth (15% vs. 8% and 7.8%, respectively; Rodgers et al., 2022). These racial inequities in child mental health and access to services occur due to the presence and effects of structural racism, rather than as a result of individual choices and behaviors (Trent et al., 2019). This is in line with a social determinants of (mental) health framework emphasizing that health outcomes are multiply determined by both upstream and downstream social conditions (World Health Organization, 2016). Indeed, Black families are more likely to engage in a number of preventative health behaviors and fewer risk behaviors yet are at greater risk for experiencing several adverse health and mental health outcomes despite these individual health promotion behaviors (Goodrum et al., 2020; Millett, 2020), highlighting the influential role of contextual factors that are shaped by structural racism.

## The Impact of Structural Racism on Youth Mental Health

Structural racism operates at multiple levels, including institutional, personally mediated, and internalized (Trent et al., 2019). Research in the mental health field has focused primarily on personally mediated or interpersonal racism (e.g., racial discrimination) and internalized racism to the exclusion of institutional forms of racism. Research on racial discrimination reveals that this form of racism is a ubiquitous reality for most BIPOC youth. For example, Black adolescents experience an average of five incidents of racial discrimination per day (English et al., 2020), and these experiences are associated with negative emotional, behavioral, and physical health outcomes (Bernard et al., 2022). Scholars recommend conceptualizing experiences of racial discrimination as a form of trauma exposure, within a culturally informed adverse childhood experiences framework (Bernard et al., 2020). These interpersonal experiences of discrimination occur within the context of additional forms of racism at other levels (e.g., policies, laws, institutions) and in the context of the historical and ongoing legacy of colonization.

Structural racism plays a role in maintaining racial inequities in child health both by (a) creating conditions and risk factors that are directly and indirectly detrimental to children’s well-being, and (b) erecting barriers to accessing prevention-focused programs and policies that could alleviate those risk factors. Structural racism touches virtually every aspect of BIPOC families’ lives, including economic conditions, housing, employment and wages, interactions with police and child protective services, voting power, experiences with the healthcare system, school quality, and neighborhood resources. Each of these interconnected domains is influential for children’s mental health. Black Americans hold only 15–20% as much net wealth as white Americans, and the racial wealth gap has been increasing over the past several years (Aladangady & Forde, 2021). Contrary to persistent stereotypes that this disparity is due to inferior work ethic or innate ability, the racial wealth gap is attributable to historical and current practices and policies, such as redlining, discriminatory lending practices, wage inequities, and unequal access to high-quality education (Beech et al., 2021). Relatedly, discriminatory housing policies have led to limited access to affordable housing, declining home ownership rates, and much greater risk of homelessness for families of color (Aladangady & Forde, 2021; Beech et al., 2021; Fusaro et al., 2018). These economic and housing conditions are directly linked with children’s mental and physical health (Van Ryzin et al., 2018).

For Black families and other families of color, interactions with authorities such as police and child protective services, though ostensibly intended to keep children and families safer, are fraught with bias, threat, and surveillance. Black children are disproportionately harmed by placement in foster care and negative effects of family separation, and this disproportionality persists due to structural racism both within the child welfare system itself and in broader society (Dettlaff & Boyd, 2020). Increasing occurrence of police brutality toward Black Americans (GBD 2019 Police Violence US Subnational Collaborators., 2021), and associated exposure to videos of these events online, has been documented to have a negative impact on children’s mental health (Tynes et al., 2019). Research documents several additional impacts of structural racism on mental health-related risk for children and families, such as the effects of chronic stress due to racial trauma and interpersonal discrimination (Saleem et al., 2020), consequences of educational inequality on academic outcomes and mental health (Fadus et al., 2021), and inequities in juvenile justice system involvement (Kang & Burton, 2014; Trent et al., 2019). These effects of structural racism are intertwined with one another, leading to compounding negative effects on children’s mental health and well-being. The comprehensive effects of structural racism on children’s well-being demand a comprehensive, prevention-oriented response.

The effects of structural racism on children’s mental health are further exacerbated by the fact that racism also erects barriers to accessing services that could ameliorate these negative impacts. One mechanism through which structural racism impedes access to prevention-focused policies and programs as well as activities and resources that support children’s well-being is residential segregation. Compared to neighborhoods where Black and Hispanic/Latinx children live, predominately white neighborhoods are more likely to have high-quality schools and daycares, playgrounds, better environmental quality, and healthy food options (Acevedo-Garcia et al., 2020), all of which are associated with children’s physical and mental health. Moreover, hospitals and health care centers—often a site of delivery for preventive interventions—are primarily located in predominately white communities (Vaughan Sarrazin et al., 2009). Thus, though BIPOC families on average may face greater need for prevention services compared to white families due to the factors outlined above, these services are far less accessible to them. There is a critical need to mitigate the impact of structural racism to improve health outcomes among youth, and prevention science is uniquely positioned to help address this critical need.

## The Need for Prevention Science to Fully Recognize and Address the Role of Structural Racism

Prevention science, with its focus on community engagement in the implementation of preventive interventions, lends itself well to promoting health equity. Its emphasis on health promotion and protective factors is also in line with a strengths-based approach that can foster resilience in families who have experienced racism-related adversity. Yet, many prevention efforts are *equity-implicit* (Gregory et al., 2016) and/or adopt a “one-size-fits-all” approach that does not explicitly address or consider structural racism. For example, a 2015 discussion paper published by the National Academy of Medicine, advocating for prevention science as a means to improve children’s mental health, set an ambitious goal of reducing racial disparities in youth behavioral health problems by 20% over a decade, but included no discussion of structural racism as a cause of these disparities or strategies to dismantle racism (Hawkins et al., 2015). Such an oversight misses the mark on identifying underlying causes of racial disparities and implies an individualized approach that may only serve to perpetuate these disparities. Even recent innovative efforts to connect prevention science with policy to address intergenerational poverty, while acknowledging racial disparities in child poverty rates, do not explicitly attend to structural racism as a driving force of intergenerational poverty (Van Ryzin et al., 2018), despite extensive evidence of its role (Beech et al., 2021). This is a missed opportunity given the higher potential impact of addressing the intertwined phenomena of poverty and racism together through prevention science. Within the child and family field, the majority of widely implemented family-based prevention programs are equity-implicit in that they target positive outcomes for all families regardless of racial, socioeconomic, or other identity background, or emphasize family-centeredness (i.e., individual fit) without considering families’ lived experiences of marginalization. There are some benefits to a universal approach, such as destigmatizing the use of mental health services or parenting support. Even when reducing disparities is not a direct goal of equity-implicit programs, these prevention approaches may at times result in reduced disparities as families with more exposure to risk factors, in some instances, benefit more (Gardner et al., 2009; Sasser et al., 2017). However, the reach, cultural relevance, and impact of these programs would be further strengthened by adopting an *equity-explicit* approach that directly considers and addresses the effects of systemic racism.

In contrast to equity-implicit or “one-size-fits-all” prevention approaches, equity-explicit, culturally responsive approaches directly aim to reduce disparities, for example by explicitly considering the effects of racism, incorporating culturally relevant protective processes, or foregrounding equity-related goals (Gregory et al., 2016; E. P. Smith et al., 2022). These approaches are in line with an *antiracism* framework that actively seeks to recognize how racism operates at multiple levels, confront racial inequities, and pursue practices and policies that lead to elimination of those inequities (Cooper et al., 2022; Roberts & Rizzo, 2021). Equity-explicit approaches are also consistent with decolonized practice, which is critical to dismantling the historical and present-day effects of colonization and requires centering Indigenous perspectives and methods. Meta-analytic findings suggest that culturally responsive behavioral health interventions have larger effects than unadapted (i.e., equity-implicit) interventions (Hall et al., 2016; Soto et al., 2018). Adopting an equity-explicit, antiracist lens in child and adolescent mental health prevention and treatment can strengthen the effectiveness and impact of those efforts and reduce access barriers. For example, prevention efforts can have a greater impact by aligning research priorities with community and patient perspectives to address issues that are of primary concern to BIPOC families; attending to unique and varied forms of oppression informed by intersectionality; removing barriers to accessing prevention-focused programming through targeted implementation strategies; addressing contextual economic and material conditions that are detrimental to children’s mental health; and leveraging existing strengths in BIPOC families and communities. Without asking research questions and applying methods that are informed and contextualized by the realities of structural racism, researchers may inadvertently reinforce racial inequities by over-individualizing problems and solutions (thereby pathologizing individuals for systemic problems) and/or overgeneralizing the population (assuming that one-size-fits-all approaches will equitably meet all families’ needs). Similarly, practitioners and other stakeholders working with children and families of color must consider the role of structural racism on children’s clinical presentations and the cultural and contextual appropriateness of “evidence-based” prevention and intervention strategies.

## Strategies for Prevention Science to Recognize and Address the Role of Structural Racism

Informed by literature in the fields of prevention science, psychology, public health, implementation science, and other related disciplines, we recommend the following practices for applying antiracist prevention science to improve child mental health equity.

1. *Establish and foster truly equitable community and patient partnerships*. Community engagement is central to conducting antiracist, culturally congruent prevention-oriented research and clinical efforts and can have wide-reaching effects on child and community health outcomes. A powerful example of effective community-based partnership, Self-Healing Communities (Porter et al., 2016), was an effort to create a comprehensive model to address widening health disparities in 42 communities in Washington state. It involved bringing together community members to generate low-cost, locally promoted, sustainable solutions that address a root cause of health disparities (i.e., adverse childhood experiences) by creating new cultural norms around health. Importantly, this approach treated all collaborating parties (i.e., funders, content experts, service providers, and community members) as partners who served as leaders in their own spheres of influence. The results of these efforts led to major community impacts, such as a 98% decrease in youth suicide, a 53% decrease in youth arrests, and an estimated 35× return on investment (Porter et al., 2016). Community engagement is a key component to enhancing health equity because it facilitates more accurate identification of root causes of health problems, garners trust and buy-in from individuals most affected by the health problems, and mobilizes community members to be agents of cultural and social change. When conducted with equity at the forefront, community engagement has the potential to reduce power imbalances between researchers and community members which allows for fuller integration of community perspectives and higher likelihood of achieving positive health outcomes for historically marginalized groups.
2. *Diversify the field through mentorship of clinicians and scholars of color focused on family well-being*. One avenue to foster research and clinical efforts that promote racial equity is to mentor individuals from diverse backgrounds and/or who conduct health equity-related research (Milburn et al., 2019). Health equity-focused mentoring programs can increase mentees’ use of culturally responsive practices, including community engagement and collaboration in developing and implementing preventive interventions to improve family well-being, and greater attention to the role of structural racism and experiences of discrimination in the lives of families. Mentoring can enhance the professional development of scholars of color to become thought leaders in the field of prevention science. These mentoring efforts can improve diversity and equity in research and clinical settings, and enhance positive mental health outcomes for diverse populations of children and families.
3. *Assemble diverse, transdisciplinary research teams and integrate multiple disciplines to generate new theories, methods, and frameworks*. A 2019 policy statement of the American Academy of Pediatrics highlighted the critical need for multidisciplinary partnerships to combat racism and its effects on child health (Trent et al., 2019). An example of leveraging transdisciplinary approaches to foster health equity can be found in the HIV literature. Efforts to end the HIV epidemic have recently been bolstered by transdisciplinary approaches integrating across implementation science, social and behavioral sciences, and community perspectives to produce new frameworks to enhance prevention and treatment of HIV (Pyra et al., 2022). Such an approach has also been suggested to address the alarming disparity in Black maternal and infant health and mortality; a disparity that exists independent of maternal education and income (Dagher & Linares, 2022). Despite decades-long recognition of this public health crisis (Singh & Yu, 2019), transdisciplinary research on the issue is still in its formative phase (Bond et al., 2021; Owens & Fett, 2019). A transdisciplinary approach can similarly be applied to the prevention of child and adolescent mental health difficulties to promote health equity in this area by integrating perspectives from multiple fields and from community stakeholders. Explicit, antiracist research agendas can be generated by establishing advisory boards and research teams made up of patients, stakeholders (e.g., providers, funders), and interdisciplinary scholars across the social and natural sciences (psychology, biology), healthcare professions (nursing, social work) and humanities (sociology, anthropology, religious studies) at the beginning conceptual stage of their research. Targeted, collaborative conceptual efforts can result in novel frameworks, such as Parker (2021)’s Afrocentric perspective on Black maternal health or a redefined, antiracist approach to child protection (Wakeman et al., 2022), that integrate systemic racism and prevention efforts in research from the outset to improve child health outcomes.
4. *Explicitly incorporate structural racism into theory, methods, frameworks, conceptualizations, and research questions*. This includes using system-centered language (Buchanan et al., 2021), describing racism (not race) as a root cause of racial health disparities, moving beyond exclusive consideration of individual and interpersonal forms of racism, considering the impact of racism on parents (stress, psychological functioning, and positive parenting), and drawing from models for radical healing (French et al., 2020). To understand the impact of systemic racism on children’s mental health, it is imperative to incorporate measurement of racism in prevention studies and evaluate the effects of preventive interventions on aspects of racism (Dean & Thorpe Jr., 2022; Neblett, 2023). Measuring racism and its impact on health is a difficult but critical task. Quantitatively, a variety of methodological approaches have been proposed (Adkins-Jackson et al., 2022; Dean & Thorpe Jr., 2022; Neblett, 2023), including using person-centered approaches to model intersections of social identities; incorporating social and contextual moderators in systematic reviews and meta-analyses; operationalizing systemic and institutional racism through system-level data (e.g., census, public health data); using index measures to simultaneously assess multiple aspects of structural and institutional racism; and applying within-group designs to understand heterogeneity within communities of color rather than comparing to a white “norm.” Furthermore, the use of qualitative and mixed methods is recommended to allow people experiencing racism to describe how it affects their lives. Utilizing qualitative and mixed methods provides rich information that allows us to better measure and understand the impact of racism on multiple levels. As one example of foregrounding equity in prevention frameworks, three national organizations (i.e., National Prevention Science Coalition; Campaign for Trauma-informed Policy and Practice; PACEs Connections) have come together to accelerate the movement to prevent trauma and foster resilience in the USA. They are guided by social justice frameworks and work to create local and national coalitions that are equity-focused and trauma-informed (National Prevention Science Coalition, 2022)
5. *Recognize and build on existing strengths that promote resilience in BIPOC families*. Much of the scholarship on minoritized children’s mental health is deficit-oriented, focusing on the challenges and higher rates of mental health disorders faced by children of color, and in particular Black children. These perspectives overlook the many existing strengths that Black and Brown families draw on to promote children’s positive development (García Coll et al., 1996). For example, traditional understandings of the impact of stress on parenting indicate that more exposure to a given stressor will lead to worse parenting quality; however, literature on Black parents’ responses to experiences of racial discrimination reveals that parents may respond resiliently to this stressor, ramping up their protective parenting in the form of ethnic-racial socialization (McNeil Smith et al., 2016). Ethnic-racial socialization and other cultural factors such as racial and ethnic identity and cultural orientation can promote BIPOC children’s healthy development across a number of domains (Anderson & Stevenson, 2019; Carlo et al., 2022) and should be integrated into family-based prevention efforts. While family-centered practice emphasizes caregivers as partners in decision making and individualized alignment of goals for children, practitioners may need additional training to understand and leverage the unique strengths of minoritized families and engage in truly family-centered practice.
6. *Attend to intersectionality and consider how structural racism interacts with stigma and oppression related to other social identities*. Prevention requires a nuanced understanding of the early risk and resilience factors that can contribute to later adverse health outcomes. It is well known that structural racism impacts groups differently based on race and other marginalized social identities (e.g., sexual orientation, ability status). The risk and resilience factors among individuals with multiple marginalized identities are different than those factors among individuals with one of these identities. This is known as intersectionality. Intersectionality theory refers to the way individuals’ experiences are shaped by the multiple social identities they hold and the interlocking, *multiplicative* forms of oppression tied to these identities (Combahee River Collective, 2000; Crenshaw, 1990). Intersectionality theory has important implications for child mental health. For example, recent data suggests that Black LGBTQ youth may be at particularly heightened risk for suicide because they experience high rates of depressive symptoms but are less likely compared to their White and/or non-LGBTQ counterparts to receive mental healthcare (Rodgers et al., 2022). This is compounded by the fact that many Black LGBTQ youth live in the Southeast U.S. (The Trevor Project, 2021)—a region with scarce mental health services (Whitney & Peterson, 2019). Recently, some have applied an intersectional lens to child mental health prevention science. For example, Opara et al. (2020) expanded the Interpersonal-Psychological Theory of Suicide to incorporate intersectionality theory including consideration of risk and protective factors across multiple contextual levels. Similar to this approach, prevention science aiming to promote children’s mental health in other areas can apply an intersectional lens to traditional family-centered practices to more effectively account for both risk and resilience factors that may uniquely affect youth experiencing multiple compounding forms of oppression related to their social identities.
7. *Use implementation science to promote access and sustainability*. Implementation science allows for the use of insights from equitable community and patient partnerships to create and rigorously evaluate targeted implementation strategies that align with priorities, values, and needs of BIPOC families. For example, the PersIn approach is an implementation strategy for behavioral parent training programs that systematically individualizes the therapy process with the goal of enhancing engagement and outcomes for families from minoritized groups (McCabe et al., 2020). Applying implementation frameworks may also help to understand and generate implementation strategies to reduce inequities. For example, rather than focusing primarily on individual-level factors to understand mental health disparities, implementation frameworks such as the Consolidated Framework for Implementation Research (CFIR) explicitly guide researchers to consider implementation strategies that address “outer context” factors, which would include systemic racism (Chinman et al., 2017). Indeed, new implementation frameworks such as the Health Equity Implementation Framework direct researchers to center and prioritize outer systemic factors over interpersonal or individual determinants, to increase effectiveness of implementation strategies in promoting equity (Woodward et al., 2019). Finally, sophisticated implementation research designs that randomize the receipt of implementation strategies at the system or agency level (e.g., cluster randomized trials) allow for rigorous evaluation of equity-focused implementation strategies (Wolfenden et al., 2021).
8. *Apply prevention science findings to advocate for health equity-related policies that promote children’s mental health, including improving economic conditions for families of color*. Prevention and implementation science can facilitate advocacy for health equity-related policies (Emmons & Chambers, 2021). Strategic policy advocacy in concert with equity-focused implementation strategies may serve to reduce health disparities given that policy changes seek to address issues at a broader (e.g., local, state, national government/system) level. These “outer context” variables point to a great need to consider policy implications of equity-focused work earlier (Damschroder et al., 2009) and to better understand how policies may serve to undermine or strengthen equity-focused prevention and child development efforts. Specific considerations may include incentivizing programs aimed at reducing disparities, ensuring that funds are being appropriately allocated to programs and interventions that seek to maximize equity in diverse communities (Murry et al., 2022), recognizing the multiplicative impact of environmental stressors on development, and greater emphasis on measuring the impact of policies addressing environmental influences (e.g., neighborhood poverty, affordable housing, healthcare access, school and educational opportunities; Komro et al., 2013). Furthermore, prevention science and implementation science must be leveraged to advocate for and support BIPOC families in accessing entitlements and other material resources when needed. Many BIPOC families who are eligible for entitlement programs, such as Temporary Assistance for Needy Families (TANF), are not accessing them, and state-level policies lead to inequitable administration of these programs by race (Hetling et al., 2021). Prevention science to promote children’s well-being must address not only barriers related to mental health care access but also the broader economic conditions created by structural racism. Without addressing these underlying economic conditions, prevention efforts are unlikely to be successful in achieving long-term equity in children’s mental health, given the persistent racial wealth gap and the detrimental effects of poverty on children’s mental health. Underscoring all of this is the need for additional research studying the impact of public policies on children’s developmental outcomes; without this, we face an uphill battle to recognizing the importance of policy to promoting longstanding equity achievement in the field of child mental health.

## Conclusion

Structural racism has contributed to persistent racial inequities in children’s mental health, despite advances in prevention science and significant efforts to promote child and family well-being. The field of prevention science is ideally positioned to help address these inequities due to its emphasis on community engagement, health promotion, and strength-based approaches. Yet, many prevention efforts are equity-implicit and do not attend to the role of racism in perpetuating and exacerbating mental health difficulties among youth of color. To move toward more effective and impactful prevention efforts and promote equity for BIPOC families, it is critical for the field to explicitly consider and address the role of structural racism in children’s mental health. By adopting antiracist strategies such as those outlined above, prevention science can more fully realize its potential in reducing inequities for BIPOC children, and promoting positive health outcomes for all children.

## References

[R1] Acevedo-Garcia D, Noelke C, McArdle N, Sofer N, Hardy EF, Weiner M, Baek M, Huntington N, Huber R, & Reece J (2020). Racial and ethnic inequities in children’s neighborhoods: Evidence from the new child opportunity index 2.0: Study uses the Child Opportunity Index 2.0 to examine geographic and racial/ethnic inequities children are exposed to in the one hundred largest metropolitan areas of the United States. Health Affairs, 39(10), 1693–1701. 33017244 10.1377/hlthaff.2020.00735

[R2] Adkins-Jackson PB, Chantarat T, Bailey ZD, & Ponce NA (2022). Measuring structural racism: A guide for epidemiologists and other health researchers. American Journal of Epidemiology, 191(4), 539–547. 10.1093/aje/kwab239 34564723 PMC9077112

[R3] Aladangady A, & Forde A (2021). Wealth inequality and the racial wealth gap. FEDS Notes. Washington: Board of Governors of the Federal Reserve System. 10.17016/2380-7172.2861. https://www.federalreserve.gov/econres/notes/feds-notes/wealth-inequality-and-the-racial-wealth-gap-20211022.html

[R4] Anderson RE, & Stevenson HC (2019). RECASTing racial stress and trauma: Theorizing the healing potential of racial socialization in families. The American Psychologist, 74(1), 63–75. 10.1037/amp0000392 30652900 PMC8807344

[R5] Beech BM, Ford C, Thorpe RJ, Bruce MA, & Norris KC (2021). Poverty, racism, and the public health crisis in America. Frontiers in Public Health, 9, 699049. 10.3389/fpubh.2021.699049 34552904 PMC8450438

[R6] Bernard DL, Calhoun CD, Banks DE, Halliday CA, Hughes-Halbert C, & Danielson CK (2020). Making the “C-ACE” for a culturally-informed adverse childhood experiences framework to understand the pervasive mental health impact of racism on Black youth. Journal of Child & Adolescent Trauma, 1–15. 10.1007/s40653-020-00319-9 33986909 PMC8099967

[R7] Bernard DL, Smith Q, & Lanier P (2022). Racial discrimination and other adverse childhood experiences as risk factors for internalizing mental health concerns among Black youth. Journal of Traumatic Stress, 35(2), 473–483. 10.1002/jts.22760 34800051 PMC9035019

[R8] Bond RM, Gaither K, Nasser SA, Albert MA, Ferdinand KC, Njoroge JN, Parapid B, Hayes SN, Pegus C, Sogade B, Grodzinsky A, Watson KE, McCullough CA, Ofili E, & Association of Black Cardiologists. (2021). Working agenda for Black mothers: A position paper from the Association of Black Cardiologists on solutions to improving Black maternal health. Circulation. Cardiovascular Quality and Outcomes, 14(2), e007643. 10.1161/CIRCOUTCOMES.120.007643 33563007 PMC7887097

[R9] Buchanan NT, Perez M, Prinstein MJ, & Thurston IB (2021). Upending racism in psychological science: Strategies to change how science is conducted, reported, reviewed, and disseminated. American Psychologist, 76(7), 1097–1112. 10.1037/amp0000905 34990171

[R10] Carlo G, Murry VM, Davis AN, Gonzalez CM, & Debreaux ML (2022). Culture-related adaptive mechanisms to race-related trauma among African American and US Latinx youth. Adversity and Resilience Science, 3(3), 247–259. 10.1007/s42844-022-00065-x 35677462 PMC9162880

[R11] Catalano RF, Fagan AA, Gavin LE, Greenberg MT, Irwin CE, Ross DA, & Shek DT (2012). Worldwide application of prevention science in adolescent health. The Lancet, 379(9826), 1653–1664. 10.1016/S0140-6736(12)60238-4 PMC4398056 22538180

[R12] Centers for Disease Control and Prevention. (2021). Media Statement from CDC Director Rochelle P. Walensky, MD, MPH, on Racism and Health. CDC. https://www.cdc.gov/media/releases/2021/s0408-racism-health.html

[R13] Chinman M, Woodward EN, Curran GM, & Hausmann LRM (2017). Harnessing implementation science to increase the impact of health equity research. Medical Care, 55(Suppl 92), S16–S23. 10.1097/MLR.0000000000000769 28806362 PMC5639697

[R14] National Prevention Science Coalition. (2022). Building a national movement to prevent trauma & foster resilience: Workshop series. https://www.npscoalition.org/prevent-trauma-workshop-series

[R15] Combahee River Collective. (2000). The Combahee River Collective Statement. In Smith B (Ed.), Home Girls: A Black Feminist Anthology. Rutgers University Press.

[R16] Cooper SM, Hurd NM, & Loyd AB (2022). Advancing scholarship on anti-racism within developmental science: Reflections on the special section and recommendations for future research. Child Development, 93(3), 619–632. 10.1111/cdev.13783 35596641

[R17] Crenshaw K (1990). Mapping the margins: Intersectionality, identity politics, and violence against women of color. Stanford Law Review, 43, 1241.

[R18] Dagher RK, & Linares DE (2022). A critical review on the complex interplay between social determinants of health and maternal and infant mortality. Children (Basel, Switzerland), 9(3), 394. 10.3390/children9030394 35327766 PMC8947729

[R19] Damschroder LJ, Aron DC, Keith RE, Kirsh SR, Alexander JA, & Lowery JC (2009). Fostering implementation of health services research findings into practice: A consolidated framework for advancing implementation science. Implementation Science, 4(1), 50. 10.1186/1748-5908-4-50 19664226 PMC2736161

[R20] Dean LT, & Thorpe RJ Jr. (2022). What structural racism is (or is not) and how to measure it: Clarity for public health and medical researchers. American Journal of Epidemiology, 191(9), 1521–1526. 10.1093/aje/kwac112 35792088 PMC9437815

[R21] Dettlaff AJ, & Boyd R (2020). Racial disproportionality and disparities in the child welfare system: Why do they exist, and what can be done to address them? The ANNALS of the American Academy of Political and Social Science, 692(1), 253–274. 10.1177/0002716220980329

[R22] Dunst CJ (2002). Family-centered practices: Birth through high school. The Journal of Special Education, 36(3), 141–149. 10.1177/00224669020360030401

[R23] Emmons KM, & Chambers DA (2021). Policy implementation science—An unexplored strategy to address social determinants of health. Ethnicity & Disease, 31(1), 133–138. 10.18865/ed.31.1.133 33519163 PMC7843047

[R24] English D, Lambert SF, Tynes BM, Bowleg L, Zea MC, & Howard LC (2020). Daily multidimensional racial discrimination among Black U.S. American adolescents. Journal of Applied Developmental Psychology, 66, 101068. 10.1016/j.appdev.2019.101068 33994610 PMC8117402

[R25] Fadus MC, Valadez EA, Bryant BE, Garcia AM, Neelon B, Tomko RL, & Squeglia LM (2021). Racial disparities in elementary school disciplinary actions: Findings from the ABCD study. Journal of the American Academy of Child & Adolescent Psychiatry, 60(8), 998–1009. 10.1016/j.jaac.2020.11.017 33359407 PMC8860403

[R26] Fishbein D (2021). The pivotal role of prevention science in this syndemic. Prevention Science, 22(1), 94–99. 10.1007/s11121-020-01180-w 33215309 PMC7676471

[R27] French BH, Lewis JA, Mosley DV, Adames HY, Chavez-Dueñas NY, Chen GA, & Neville HA (2020). Toward a psychological framework of radical healing in communities of color. The Counseling Psychologist, 48(1), 14–46. 10.1177/0011000019843506

[R28] Fusaro VA, Levy HG, & Shaefer HL (2018). Racial and ethnic disparities in the lifetime prevalence of homelessness in the United States. Demography, 55(6), 2119–2128. 10.1007/s13524-018-0717-0 30242661 PMC7665902

[R29] García Coll C, Lamberty G, Jenkins R, McAdoo HP, Crnic K, Wasik BH, & García HV (1996). An integrative model for the study of developmental competencies in minority children. Child Development, 67(5), 1891–1914. 10.2307/1131600 9022222

[R30] Gardner F, Connell A, Trentacosta CJ, Shaw DS, Dishion TJ, & Wilson MN (2009). Moderators of outcome in a brief family-centered intervention for preventing early problem behavior. Journal of Consulting and Clinical Psychology, 77, 543–553. 10.1037/a0015622 19485594 PMC2793096

[R31] GBD Police Violence US Subnational Collaborators 2019. (2021). Fatal police violence by race and state in the USA, 1980–2019: A network meta-regression. The Lancet, 398(10307), 1239–1255. 10.1016/S0140-6736(21)01609-3 PMC8485022 34600625

[R32] Gertler P, Heckman J, Pinto R, Zanolini A, Vermeersch C, Walker S, Chang SM, & Grantham-McGregor S (2013). Labor market returns to early childhood stimulation: A 20-year followup to an experimental intervention in Jamaica (Working Paper No. 19185). National Bureau of Economic Research. 10.3386/w19185

[R33] Goodrum NM, & Prinz RJ (2022). Family-based prevention of child traumatic stress. Pediatric Clinics of North America, 69(4), 633–644. 10.1016/j.pcl.2022.04.011 35934490 PMC9554837

[R34] Goodrum NM, Smith DW, Hanson RF, Moreland AD, Saunders BE, & Kilpatrick DG (2020). Longitudinal relations among adolescent risk behavior, family cohesion, violence exposure, and mental health in a national sample. Journal of Abnormal Child Psychology, 48(11), 1455–1469. 10.1007/s10802-020-00691-y 32845455 PMC7530104

[R35] Gregory A, Hafen CA, Ruzek E, Mikami AY, Allen JP, & Pianta RC (2016). Closing the racial discipline gap in classrooms by changing teacher practice. School Psychology Review, 45(2), 171–191. 10.17105/SPR45-2.171-191 28190913 PMC5302858

[R36] Hall GCN, Ibaraki AY, Huang ER, Marti CN, & Stice E (2016). A meta-analysis of cultural adaptations of psychological interventions. Behavior Therapy, 47(6), 993–1014. 10.1016/j.beth.2016.09.005 27993346

[R37] Hawkins JD, Jenson JM, Catalano R, Fraser MW, Botvin GJ, Shapiro V, Brown CH, Beardslee W, Brent D, Leslie LK, Rotheram-Borus MJ, Shea P, Shih A, Anthony E, Haggerty KP, Bender K, Gorman-Smith D, Casey E, & Stone S (2015). Unleashing the power of prevention. NAM Perspectives. 10.31478/201506c

[R38] Hetling A, Holcomb S, Seith D, Riordan A, Santiago J, Roman JL, Lupinacci S, & Seehra A (2021). An equity analysis of applying for welfare: TANF application and denial reasons by household and county characteristics. Social Service Review, 95(4), 616–651. 10.1086/717298

[R39] Kang H-K, & Burton DL (2014). Effects of racial discrimination, childhood trauma, and trauma symptoms on juvenile delinquency in African American incarcerated youth. Journal of Aggression, Maltreatment & Trauma, 23(10), 1109–1125. 10.1080/10926771.2014.968272

[R40] Komro KA, Tobler AL, Delisle AL, O’Mara RJ, & Wagenaar AC (2013). Beyond the clinic: Improving child health through evidence-based community development. BMC Pediatrics, 13(1), 172. 10.1186/1471-2431-13-172 24138839 PMC4016148

[R41] McCabe KM, Yeh M, & Zerr AA (2020). Personalizing behavioral parent training interventions to improve treatment engagement and outcomes for culturally diverse families. Psychology Research and Behavior Management, 13, 41–53. 10.2147/PRBM.S230005 32021508 PMC6966146

[R42] McNeil Smith S, Reynolds JE, Fincham FD, & Beach SRH (2016). Parental experiences of racial discrimination and youth racial socialization in two-parent African American families. Cultural Diversity & Ethnic Minority Psychology, 22(2), 268–276. 10.1037/cdp0000064 26237542

[R43] Metzger IW, Anderson RE, Are F, & Ritchwood T (2021). Healing interpersonal and racial trauma: Integrating racial socialization into trauma-focused cognitive behavioral therapy for African American youth. Child Maltreatment, 26(1), 17–27. 10.1177/1077559520921457 32367729 PMC8807349

[R44] Milburn NG, Hamilton AB, Lopez S, & Wyatt GE (2019). Mentoring the next generation of behavioral health scientists to promote health equity. American Journal of Orthopsychiatry, 89(3), 369–377. 10.1037/ort0000415 31070422 PMC7577403

[R45] Millett GA (2020). New pathogen, same disparities: Why COVID-19 and HIV remain prevalent in U.S. communities of colour and implications for ending the HIV epidemic. Journal of the International AIDS Society, 23(11), e25639. 10.1002/JIA2.25639 33222424 PMC7645849

[R46] Murry VM, Bradley C, Cruden G, Brown CH, Howe GW, Sepùlveda M-J, Beardslee W, Hannah N, & Warne D (2022). Re-envisioning, retooling, and rebuilding prevention science methods to address structural and systemic racism and promote health equity. Prevention Science. 10.1007/s11121-022-01439-4 PMC9554395 36223046

[R47] Neblett EW (2023). Racism measurement and influences, variations on scientific racism, and a vision. Social Science & Medicine, 316, 115247. 10.1016/j.socscimed.2022.115247 36180279

[R48] Opara I, Assan MA, Pierre K, Gunn JF, Metzger I, Hamilton J, & Arugu E (2020). Suicide among Black children: An integrated model of the Interpersonal-Psychological Theory of Suicide and intersectionality theory for researchers and clinicians. Journal of Black Studies, 51(6), 611–631. 10.1177/0021934720935641 34305168 PMC8301214

[R49] Owens DC, & Fett SM (2019). Black maternal and infant health: Historical legacies of slavery. American Journal of Public Health, 109(10), 1342–1345. 10.2105/AJPH.2019.305243 31415204 PMC6727302

[R50] Parker A (2021). Reframing the narrative: Black maternal mental health and culturally meaningful support for wellness. Infant Mental Health Journal, 42(4), 502–516. 10.1002/imhj.21910 33470438

[R51] Patterson GR, Forgatch MS, & DeGarmo DS (2010). Cascading effects following intervention. Development and Psychopathology, 22(4), 949–970. 10.1017/S0954579410000568 20883592 PMC2965055

[R52] Porter L, Martin K, & Anda R (2016). Self-healing communities. Robert Wood Johnson Foundation https://www.rwjf.org/en/library/research/2016/06/self-healing-communities.html

[R53] Pyra M, Motley D, & Bouris A (2022). Moving toward equity: Fostering transdisciplinary research between the social and behavioral sciences and implementation science to end the HIV epidemic. Current Opinion in HIV and AIDS, 17(2), 89–99. 10.1097/COH.0000000000000726 35225249

[R54] Ramchand R, Gordon JA, & Pearson JL (2021). Trends in suicide rates by race and ethnicity in the United States. JAMA Network Open, 4(5), e2111563. 10.1001/jamanetworkopen.2021.11563 34037735 PMC8155821

[R55] Roberts SO, & Rizzo MT (2021). The psychology of American racism. American Psychologist, 76(3), 475–487. 10.1037/amp0000642 32584061

[R56] Rodgers CRR, Flores MW, Bassey O, Augenblick JM, & Cook BL (2022). Racial/ethnic disparity trends in children’s mental health care access and expenditures from 2010-2017: Disparities remain despite sweeping policy reform. Journal of the American Academy of Child & Adolescent Psychiatry, 61(7), 915–925. 10.1016/j.jaac.2021.09.420 34627995 PMC8986880

[R57] Saleem FT, Anderson RE, & Williams M (2020). Addressing the “Myth” of racial trauma: Developmental and ecological considerations for youth of color. Clinical Child and Family Psychology Review, 23(1), 1–14. 10.1007/s10567-019-00304-1 31641920 PMC8845073

[R58] Sandler I, Ingram A, Wolchik S, Tein JY, & Winslow E (2015). Long-term effects of parenting-focused preventive interventions to promote resilience of children and adolescents. Child Development Perspectives, 9(3), 164–171. 10.1111/cdep.12126 30854024 PMC6407875

[R59] Sasser TR, Bierman KL, Heinrichs B, & Nix RL (2017). Preschool intervention can promote sustained growth in the executive-function skills of children exhibiting early deficits. Psychological Science, 28(12), 1719–1730. 10.1177/0956797617711640 29065281 PMC5725267

[R60] Singh GK, & Yu SM (2019). Infant mortality in the United States, 1915-2017: Large social inequalities have persisted for over a century. International Journal of Maternal and Child Health and AIDS, 8(1), 19–31. 10.21106/ijma.271 31049261 PMC6487507

[R61] Smith EP, Yzaguirre MM, Dwanyen L, & Wieling E (2022). Culturally relevant parenting approaches among African American and Latinx children and families: Toward resilient, strengths-based, trauma-informed practices. Adversity and Resilience Science, 3(3), 209–224. 10.1007/s42844-022-00059-9

[R62] Soto A, Smith TB, Griner D, Domenech Rodríguez M, & Bernal G (2018). Cultural adaptations and therapist multicultural competence: Two meta-analytic reviews. Journal of Clinical Psychology, 74(11), 1907–1923. 10.1002/jclp.22679 30091201

[R63] The Trevor Project. (2021). Research brief: LGBTQ Youth in the South (pp. 1–4).

[R64] Trent M, Dooley DG, Dougé J, Section on adolescent health, council on community pediatrics, committee on adolescence, Cavanaugh RM Jr., , Lacroix AE, Fanburg J, Rahmandar MH, Hornberger LL, Schneider MB, Yen S, Chilton LA, Green AE, Dilley KJ, Gutierrez JR, Duffee JH, Keane VA, (2019). The impact of racism on child and adolescent health. Pediatrics, 144(2), e20191765. 10.1542/peds.2019-1765 31358665

[R65] Tynes BM, Willis HA, Stewart AM, & Hamilton MW (2019). Race-related traumatic events online and mental health among adolescents of color. Journal of Adolescent Health, 65(3), 371–377. 10.1016/j.jadohealth.2019.03.006 31196779

[R66] Van Ryzin MJ, Fishbein D, & Biglan A (2018). The promise of prevention science for addressing intergenerational poverty. Psychology, Public Policy, and Law, 24(1), 128–143. 10.1037/law0000138 PMC5933861 29731600

[R67] Vaughan Sarrazin MS, Campbell ME, Richardson KK, & Rosenthal GE (2009). Racial segregation and disparities in health care delivery: Conceptual model and empirical assessment. Health Services Research, 44(4), 1424–1444. 10.1111/j.1475-6773.2009.00977.X 19467026 PMC2739036

[R68] Wakeman SE, Bryant A, & Harrison N (2022). Redefining child protection: Addressing the harms of structural racism and punitive approaches for birthing people, dyads, and families affected by substance use. Obstetrics and Gynecology, 140(2), 167–173. 10.1097/A0G.0000000000004786 35852265

[R69] Whitney DG, & Peterson MD (2019). US national and state-level prevalence of mental health disorders and disparities of mental health care use in children. JAMA Pediatrics, 173(4), 389. 10.1001/jamapediatrics.2018.5399 30742204 PMC6450272

[R70] Williams DR, Lawrence JA, & Davis BA (2019). Racism and health: Evidence and needed research. Annual Review of Public Health, 40(1), 105–125. 10.1146/annurev-publhealth-040218-043750 PMC6532402 30601726

[R71] Wolfenden L, Foy R, Presseau J, Grimshaw JM, Ivers NM, Powell BJ, Taljaard M, Wiggers J, Sutherland R, Nathan N, Williams CM, Kingsland M, Milat A, Hodder RK, & Yoong SL (2021). Designing and undertaking randomised implementation trials: Guide for researchers. BMJ (Clinical Research Ed.), 372, m3721. 10.1136/bmj.m3721 PMC7812444 33461967

[R72] Woodward EN, Matthieu MM, Uchendu US, Rogal S, & Kirchner JE (2019). The health equity implementation framework: Proposal and preliminary study of hepatitis C virus treatment. Implementation Science, 14(1), 26. 10.1186/s13012-019-0861-y 30866982 PMC6417278

[R73] World Health Organization. (2016). Social determinants of health: Key concepts. World Health Organization https://www.who.int/news-room/questions-and-answers/item/social-determinants-of-health-key-concepts

